# Impact of socioeconomic status on cancer incidence and stage at diagnosis: selected findings from the surveillance, epidemiology, and end results: National Longitudinal Mortality Study

**DOI:** 10.1007/s10552-008-9256-0

**Published:** 2008-11-12

**Authors:** Limin X. Clegg, Marsha E. Reichman, Barry A. Miller, Benjamin F. Hankey, Gopal K. Singh, Yi Dan Lin, Marc T. Goodman, Charles F. Lynch, Stephen M. Schwartz, Vivien W. Chen, Leslie Bernstein, Scarlett L. Gomez, John J. Graff, Charles C. Lin, Norman J. Johnson, Brenda K. Edwards

**Affiliations:** Office of Healthcare Inspections, Office of Inspector General (54AA), U.S. Department of Veterans Affairs, 810 Vermont Ave., NW, Washington, DC 20420, USA; Surveillance Research Program, Division of Cancer Control and Population Sciences, National Cancer Institute, National Institutes of Health, Bethesda, MD, USA; Information Management Services, Inc., Silver Spring, MD, USA; Health Resources and Services Administration, Rockville, MD, USA; School of Medicine, Monash University, Melbourne, VIC, Australia; Cancer Research Center, University of Hawaii, Honolulu, HI, USA; State Health Registry of Iowa, University of Iowa, Iowa City, IA, USA; Program in Epidemiology, Division of Public Health Sciences, Fred Hutchinson Cancer Research Center, Seattle, WA, USA; Louisiana Tumor Registry, LSU School of Public Health, New Orleans, LA, USA; Department of Population Sciences, City of Hope National Medical Center/Beckman Research Institute, Duarte, CA, USA; Northern California Cancer Center, Fremont, CA, USA; Karmanos Cancer Institute, Wayne State University, Detroit, MI, USA; U.S. Bureau of the Census, Suitland, MD, USA; Surveillance Research Program, Division of Cancer Control and Population Sciences, National Cancer Institute, National Institutes of Health, Bethesda, MD, USA

**Keywords:** SEER, NLMS, Cancer incidence, Stage, Education, Income, Poverty, Unemployment, SES, Race/ethnicity, Rural/urban, Health disparities, Record linkage

## Abstract

**Background:**

Population-based cancer registry data from the Surveillance, Epidemiology, and End Results (SEER) Program at the National Cancer Institute (NCI) are mainly based on medical records and administrative information. Individual-level socioeconomic data are not routinely reported by cancer registries in the United States because they are not available in patient hospital records. The U.S. representative National Longitudinal Mortality Study (NLMS) data provide self-reported, detailed demographic and socioeconomic data from the Social and Economic Supplement to the Census Bureau's Current Population Survey (CPS). In 1999, the NCI initiated the SEER-NLMS study, linking the population-based SEER cancer registry data to NLMS data. The SEER-NLMS data provide a new unique research resource that is valuable for health disparity research on cancer burden. We describe the design, methods, and limitations of this data set. We also present findings on cancer-related health disparities according to individual-level socioeconomic status (SES) and demographic characteristics for all cancers combined and for cancers of the lung, breast, prostate, cervix, and melanoma.

**Methods:**

Records of cancer patients diagnosed in 1973–2001 when residing 1 of 11 SEER registries were linked with 26 NLMS cohorts. The total number of SEER matched cancer patients that were also members of an NLMS cohort was 26,844. Of these 26,844 matched patients, 11,464 were included in the incidence analyses and 15,357 in the late-stage diagnosis analyses. Matched patients (used in the incidence analyses) and unmatched patients were compared by age group, sex, race, ethnicity, residence area, year of diagnosis, and cancer anatomic site. Cohort-based age-adjusted cancer incidence rates were computed. The impact of socioeconomic status on cancer incidence and stage of diagnosis was evaluated.

**Results:**

Men and women with less than a high school education had elevated lung cancer rate ratios of 3.01 and 2.02, respectively, relative to their college educated counterparts. Those with family annual incomes less than $12,500 had incidence rates that were more than 1.7 times the lung cancer incidence rate of those with incomes $50,000 or higher. Lower income was also associated with a statistically significantly increased risk of distant-stage breast cancer among women and distant-stage prostate cancer among men.

**Conclusions:**

Socioeconomic patterns in incidence varied for specific cancers, while such patterns for stage were generally consistent across cancers, with late-stage diagnoses being associated with lower SES. These findings illustrate the potential for analyzing disparities in cancer outcomes according to a variety of individual-level socioeconomic, demographic, and health care characteristics, as well as by area measures available in the linked database.

## Introduction

Despite advances in knowledge concerning risk factor reduction and improvements in early detection and treatment for several cancers, socioeconomic inequalities persist in cancer incidence, morbidity, mortality, and survival [1-3]. In some instances, such inequalities may even be widening [4]. The disparities in cancer burden among racial and ethnic minorities and other disadvantaged groups prompted congressional legislation (Public Law 104-208 in 1997) mandating a review of the research programs at the National Institutes of Health (NIH) by the Institute of Medicine (IOM). The IOM report [5] was published in 1999 and was followed by Congressional legislation in 2000 (Public Law 106-525) requesting the establishment of the NIH National Center for Minority Health and Health Disparities and a strategic plan in health disparities research. In its 2006 review [6] of the Strategic Plan, the IOM study committee recommended NIH research priority areas “should include, first, the development and refinement of valid measures of exposure relevant to understanding and evaluating health disparities.” As an example, it specifically called for, “the inclusion of information on racial and ethnic subpopulations and other relevant characteristics, such as immigrant status, language preference, and detailed socioeconomic data” in population-based studies.

Population-based cancer registry data from the Surveillance, Epidemiology, and End Results (SEER) Program at the National Cancer Institute (NCI) are generally the authoritative source of data for describing disparities in cancer burden among racial/ethnic groups. However, these data are mainly based on medical records and administrative information, and thus lack individual-level data on socioeconomic status (SES). Socio-demographic information on individual cancer patients in the NCI's SEER database is limited to age, sex, race/ethnicity [7], marital status, and place of birth and residence. Key measures of individual socioeconomic status (SES), such as educational attainment, occupation, income, and employment status are not available. Data on current health status, co-morbidity, health care access, and health-risk behaviors, such as cigarette smoking, are also lacking. Consequently, socioeconomic analyses of surveillance data on cancer incidence, disease stage, treatment, and patient survival in the U.S. have generally relied on more readily available aggregate ecological data [8, 9]. To overcome the absence of individual-level SES data in cancer registries, and to provide a unique research resource that can be used for describing disparities in cancer burden, in 1999, the NCI initiated the SEER-NLMS project, linking population-based SEER cancer registry data to that from the U.S. representative National Longitudinal Mortality Study (NLMS). The NLMS provides self-reported, detailed demographic and socioeconomic data from the Social and Economic Supplement to the Census Bureau's Current Population Survey (CPS). The objective of this record linkage project was to supplement the socioeconomic information on SEER cancer patients and to assess differentials in cancer incidence, tumor characteristics, and patient survival, based on self-reported race/ethnicity, marital status, educational attainment, income, occupation, industry, employment status, nativity/immigrant status, smoking status, health status, and availability of health insurance [10, 11].

This paper presents some initial findings that pertain to the identification of health disparities from this unique database, including cancer disparities according to individual-level socioeconomic status and demographic characteristics for all cancers combined and for cancers of the lung, breast, prostate, cervix, and melanoma. In addition, the linked database itself is described including an overview of its structure, the record linkage methodology used to create it, data confidentiality issues, the representativeness of the cancer data, and its analytic potential for research.

## Materials and methods

### The Surveillance, Epidemiology, and End Results Program

Begun in 1973, the NCI SEER Program is a population-based cancer registration program, which identifies all primary cancers occurring in residents of defined geographic regions. Cancer registries of the SEER Program currently cover approximately 26% of the U.S. population. SEER collects detailed data on patient demographics, tumor characteristics, and initial therapy, and maintains follow-up of all registered patients for vital status in order to provide statistics on cancer patient survival [12]. The primary sources of SEER data are hospital medical records, pathology and radiotherapy reports, outpatient surgical center records, death certificates, and other routinely collected administrative and health records available to each registry. Quality control has been an integral part of the SEER Program since its inception [13]. Annual studies are conducted in SEER registries to evaluate the quality and completeness of the data being reported.

### The Current Population Survey and National Longitudinal Mortality Study

The CPS is a monthly survey of about 50,000 households conducted by the U.S. Bureau of the Census for the Bureau of Labor Statistics. It is the primary source of information on the labor force and demographic characteristics of the U.S. population between decennial censuses. CPS samples are selected to represent the U.S. civilian non-institutional population. Respondents are interviewed either by telephone or in-person to obtain information about the employment status of each member of the household who is 15 years of age or older [14]. In March, the Annual Social and Economic Supplement (named the Annual Demographic Survey Supplement before 2003) of CPS collects in-depth information on income and a variety of demographic characteristics. Response is higher in CPS than in many other surveys. For example, the non-response rate for the March 2002 basic CPS was 8.3% and the non-response rate for the March supplement was an additional 8.6%, which amounted to a total 2002 supplement response rate of 83.8% [15].

The NLMS is an on-going mortality follow-up study of selected cohorts of CPS respondents and the 1980 E sample (a post-enumeration sample used to measure the undercount of the 1980 Decennial Census). Currently, it contains 26 cohorts: one from the 1980 E sample and 25 from CPS, totaling approximately 2.4 million people. The 25 CPS cohorts in the NLMS were sampled between 1973 and 1998, and their surveys were conducted in March 1973, February 1978, March 1979, April 1980, August 1980, December 1980, September 1985, and for each March in the period 1981–1998. The NLMS study combined the self-reported data with death certificate information to identify mortality status and cause of death for its 26 cohorts, for the purpose of studying the effects of demographic and socioeconomic characteristics on U.S. mortality rates [16].

### The SEER-NLMS study

The SEER-NLMS study consists of identifying and matching SEER cancer patient records to NLMS records. Records for cancer patients diagnosed between 1973 and 2001 and reported to 11 SEER registries were matched to the 26 NLMS cohorts. The 11 participating SEER registries included the states of Connecticut (1973–2001 data), Hawaii (1973–2001), Iowa (1973–2001), Kentucky (1995–2001), Louisiana (1988–2001), and Utah (1973–2001); the metropolitan areas of Detroit (1973–2000), Los Angeles (1988–2001), Northern California (1973–2001 data that include the top 20 primary cancer sites for Greater Bay Area including San Francisco, Oakland, San Jose, and Monterey regions), and Seattle (1974–2001); and Greater California (the state of California excluding Los Angeles and Northern California; 1988–2001 data). Each participating SEER registry obtained approval from the appropriate institutional review board prior to the linkage.

The algorithm used to match SEER records to the CPS self-reports in the NLMS was derived directly from the two-step process to identify mortality in the NLMS [17] using personal identifiers: social security number (SSN), name (first and last), and date of birth (month and year). The first step consisted of the application of a computer-scoring algorithm to identify clearly true and clearly false matches by comparing a SEER patient's record with an NLMS record. A pair agreeing on SSN was identified as a deterministic match and considered as a true match if name and birth date also agreed. Pairs that did not agree on SSN were identified as a probabilistic match if the pair agreed on name and birth date. Probabilistic matches were scored for agreement on name, year of birth, as well as variations of demographic variables such as sex, race, and place of residence. If the agreement score exceeded an upper cut-off value, the match was considered to be true. If the agreement score was below the lower cut-off value the pair was not a match. Upper and lower cut-off values of the computer algorithm were derived empirically using two databases for which manual decisions were made in advance for all pairs. The questionable matched-pairs consisted of those deterministic matches that disagreed in either sex or birth date or those probabilistic matches with a score in the middle range. In the second step, all questionable matched-pairs were judged in a manual review by a panel of three judges operating independently to decide the final outcome of true match or false match where all information on the SEER and the NLMS records was compared for agreement. An independent verification of the validity of the NLMS matching algorithm has been conducted [18] on an American Cancer Society database.

The SEER-NLMS record matching was conducted by the Census Bureau on its premises. The matched SEER-NLMS data are kept on the premises of Census Bureau and are protected by the statutory confidentiality authority of the Census Bureau, Sect. 9 of Title 13 [19]. In all, 2.4 million NLMS records from the 25 CPS and the Census E sample were compared with 4,172,139 cancer patient records in 11 SEER registries, generating 26,844 patient matches. Of these matched patients, 2,663 patients were diagnosed with more than one primary cancer, resulting in a total of 29,883 primary cancers diagnosed during the period 1973–2001.

Of the 26,844 matched patients, we excluded 146 patients whose CPS survey data were incomplete and would not have been eligible for inclusion in the NLMS study. A small number of cancer patients were identified in records from more than one SEER registry (*n* = 106) and were excluded from the study. Because the 1980 Census E sample lacked socioeconomic information and its cohort was excluded from this study, we also excluded 1,337 patients whose SEER medical records were matched to this sample. We excluded 345 matched patients who were under 25 years of age at the time of their survey under the rationale that their reported family income was more likely reflective of their parents' rather than their own. Thus, we limited our study to the individuals who were 25 years of age or older at the time of their survey. In addition, we excluded 3,369 patients whose cancer was diagnosed before their survey and 1,392 patients who had been diagnosed with only non-invasive cancers. Hence, 20,149 matched patients were eligible for inclusion in this study.

For the cancer incidence part of the analysis (Tables 2, 3, 4, 5), an additional 8,685 matched patients were excluded. This included 3,334 patients whose SEER records were matched to the March 1973 and February 1978 CPS cohorts (because they lack follow-up information for vital status), 2,356 matched patients who were residents of one SEER registry territory at time of their CPS survey but diagnosed in another SEER area, and 2,995 patients whose cancers were diagnosed after 1998 because the NLMS mortality follow-up for the cohorts ended by 12/31/1998. Hence, 11,464 matched patients were included for the incidence analyses. Analyses on late-stage diagnoses (Table 6) are based on 15,357 patients, after excluding the 4,792 cancer patients lacking information on tumor stage from the 20,149 eligible patients.

### Demographic, socioeconomic, and other variables

All demographic and socioeconomic variables used in this analysis are from survey self-reports, except age at diagnosis, stage at diagnosis, and sex (for matched cancer cases), which are from SEER data. Therefore, for the incidence analyses, the sex variable came from NLMS for those survey participants who did not have a cancer diagnosed as of December 31, 1998, i.e., their survey record did not link to SEER database prior to this date. For late-stage diagnosis analyses, the sex variable is from SEER data.

Race and ethnic variables were categorized as non-Hispanic white, non-Hispanic black, American Indian or Alaska Native (AI/AN), Asian or Pacific Islander (API), Hispanic with its two subcategories of Mexican Hispanic and Other Hispanic, and Other or Unknown. The “Other or Unknown” category grouped all racial and ethnic categories other than the categories specified above, including those patients with missing race or ethnicity data. Marital status was classified as married, widowed, divorced/separated, never married, and unknown status. Place of residence at the time of the survey was classified into urban, rural, and unknown based on the definitions from the 1970 census (CPS cohorts 1973–1985), the 1980 census (CPS cohort 1986–1993), or the 1990 census (CPS cohorts 1994–1998) [20, 21].

Educational attainment was grouped into four categories by years of education: less than high school (<12 years), high school graduate (12 years), some post high school education (13–15 years), college education or beyond (16 years or more), and unknown. Family income refers to the total combined income of all family members during the 12 months preceding the survey and it was adjusted to 1990 dollars for inflation for individuals from different NLMS cohorts. The 1989 [22] median family income in the US was $35,255 with the poverty threshold of $12,674 for a four-person family. Thus, we categorized family income as <$12,500, $12,500–$24,999, $25,000–$34,999, $35,000–$49,999, $50,000 or more, and unknown. The poverty status for all individuals in the database was measured as of the 1990 census in terms of the ratio of the family income to the poverty threshold for a four-person family and grouped into ≤100%, 100 to <200%, 200 to <400%, 400 to <600%, and 600% or above.

Employment status was determined on the basis of employment activity during the week prior to the survey and was classified into five categories for the present analysis: employed, unemployed (seeking work during the past 4 weeks), retired, unable to work (long-term physical or mental disability), and outside the labor force (consisting of homemakers and those in school) [10]. Employment sector was defined for those employed and included the following groupings: government (federal, state, local), private, and self-employed.

Late stage is defined as the distant stage of cancer presentation at the time of diagnosis by the SEER Historical Staging scheme. Distant-stage cancer indicates that cancers have spread from the organ/site of origin to distant sites.

### Statistical analysis

Incidence analyses were conducted for all cancers combined and for six major cancers separately: lung and bronchus, colon/rectum, breast, prostate, uterine cervix, and melanoma of the skin. Age-specific cancer incidence rates were calculated by dividing the number of cancer patients in each 5-year age group by the follow-up time (in person-years) accumulated for that age group of survey participants. These age-specific rates were then age-adjusted by the direct method using the age composition of the 2000 U.S. standard population (Census p25-1130). Follow-up time for each individual started from the CPS survey date up until the date of the underlying cancer diagnosis, loss to follow-up (available only for matched patients), death, or end of study (12/1998), whichever occurred first. It was accumulated into different age groups as the individual aged. In computing the incidence rates for all cancers combined, only the first primary cancer diagnosed in a patient was counted, regardless of the cancer site, and follow-up time was allowed to accumulate only until the date of diagnosis of that first cancer. When computing the incidence rate for a specific cancer, such as female breast cancer, only the first primary breast cancer occurring in a patient was considered and the follow-up time contribution for that individual stopped at the date of diagnosis of that first breast cancer although the patient might have been diagnosed with another cancer prior to her breast cancer diagnosis.

Adjusted incidence rate ratios (i.e., hazard ratio) and their 95% confidence intervals were derived using Cox regression models that stratified baseline risks of cancer diagnosis by NLMS cohort and by their age at the survey. The six age strata used were: 25–34, 35–44, 45–54, 55–64, 65–74, and 75 years or older. Follow-up times were recoded in months.

To analyze disparities in the likelihood of late- or distant-stage diagnoses for colorectal, prostate, and breast cancer, logistic regression models adjusting for age at diagnosis (25–54, 55–64, 65–74, and 75+ years), period of diagnosis (1973–1989, 1990–1994, and 1995–2001), and SEER registry were used. Results of the late-stage diagnosis analyses are presented as adjusted odds ratios with their corresponding 95% confidence intervals. All analyses were performed using SAS statistical software (SAS Institute, Inc., Cary, North Carolina). All statistical tests are two-sided and the level of statistical significance is 0.05.

## Results

### Representativeness of matched cancer cases included for study

Table 1 compares the distribution of selected characteristics among matched SEER-NLMS patients that were included in the incidence analysis with that for the full SEER registry case file originally submitted for matching. Due to the large size of the study population, comparisons within each category of characteristics (age group, sex, etc.) were statistically significant. The magnitude of most of the differences, however, is small, and thus likely not of practical importance. Men are slightly over-represented among matched cases included in these analyses. While whites form essentially the same percentage of submitted and included cases, blacks are underrepresented and Asian/Pacific Islanders are over-represented in included cases. The percentages of non-Hispanic whites and Hispanics included in the incidence analysis are similar to those for the originally submitted cases. Differences in years of diagnosis reflect the higher likelihood to be matched to NLMS cohorts for patients diagnosed in later years than those diagnosed in earlier years. Overall, the magnitude of the differences is small and the population of patients included in these analyses can be considered to be reasonably representative of the total SEER patient population from which they were drawn.

### Selected findings on individual-level SES disparities in cancer

#### Differentials in cancer incidence

Tables 2, 3, 4, 5 show site-specific cancer incidence counts, age-adjusted rates, standard errors, rate ratios, and corresponding 95% confidence intervals, by race/ethnicity, educational attainment, family income, poverty status, employment status, employment sector, marital status, and rural/urban residence. Although data are provided for all cancers combined for the purpose of showing how the total cancer incidence burden varies by SES characteristics, the emphasis is placed on interpreting SES disparities in incidence of specific cancers, as they are likely to reveal important clues regarding cancer etiology and the distribution of risk factors by measures of socioeconomic status.

There were consistent gradients in incidence rates for major cancers such as lung, female breast, prostate, cervix, and melanoma of the skin by self-reported educational attainment, family income, and poverty status. For example, during 1979–1998, men with less than a high school education and those with a high school education had lung cancer rate ratios of 3.01 and 2.32, respectively, compared to their college-educated counterparts (Table 3). Educational gradients in lung cancer for women were smaller than those for men. Women with less than a high school education and those with a high school diploma had lung cancer rate ratios of 2.02 and 1.74 comparing to women with at least a college degree. For prostate and female breast cancers (Table 4), higher educational attainment was associated with higher cancer incidence. Compared to their college-educated counterparts, men and women with less than a high school education had rate ratios of 0.79 and 0.74 for prostate and breast cancer incidence, respectively. Educational differences in colorectal cancer were small but statistically significant, with those with a high school education or less having a rate of 1.45 times of that with a college education. Educational differentials in melanoma of the skin and cervical cancer were significant although numbers of cases are much smaller than for cancer sites described above (Table 5). Compared to those with a college education, those with less than high school education had a reduced risk for melanoma of the skin (rate ratio = 0.55), but an elevated risk for cervical cancer (rate ratio = 3.24).

Income gradients in male and female lung cancer incidence were significant (Table 3), with those with family incomes less than $12,500 having an incidence rate more than 1.7 times that of those with family incomes of $50,000 or more. The income gradient for prostate cancer (Table 4) incidence shows men with lower incomes at reduced risk relative to those with a family income of $50,000 or more. An income gradient was also observed for melanoma of the skin. Those with family incomes less than $12,500 and $12,500–$24,999 had rate ratios of 0.59 and 0.88, respectively, relative to those with a family income of $50,000 or more. There were substantial gradients for both income and poverty in cervical cancer incidence. Women at or below 100% and 100–200% of the poverty rate had cervical cancer rates of 4.30 and 3.35, respectively, higher than those with family incomes exceeding 600% of the poverty threshold.

Substantial racial/ethnic variations in incidence rates are noted for all cancers combined as well for the specific cancers examined (Tables 2, 3, 4). Compared to non-Hispanic whites, Hispanics and Asian/Pacific Islanders had significantly lower incidence rates for all cancers combined as well as for several other cancers. Specifically, compared to non-Hispanic whites, Mexicans had a lower overall cancer rate (rate ratio = 0.73), lower rates of lung cancer (male rate ratio = 0.55, female rate ratio = 0.25), and a lower rate of female breast cancer (rate ratio = 0.73). Compared to non-Hispanic whites, Asian/Pacific Islanders had a lower rate for overall cancer rate (rate ratio = 0.74), male lung cancer (rate ratio = 0.65), female lung cancer (rate ratio = 0.56), colorectal cancer (rate ratio = 0.77), prostate cancer (rate ratio = 0.59), and female breast cancer (rate ratio = 0.82). Compared to non-Hispanic white men, non-Hispanic black men had a higher overall cancer rate (rate ratio = 1.49), with higher rates of lung cancer (rate ratio = 1.73), and prostate cancer (rate ratio = 1.87), while non-Hispanic black women had a higher rate of cervical cancer (rate ratio = 2.00) relative to non-Hispanic white women. Colorectal cancer rates were also higher among non-Hispanic blacks (rate ratio = 1.44).

Tables 2, 3, 4, 5 also show site-specific incidence rates and rate ratios by marital status, employment status, employment sector/class of worker, and rural/urban residence. Worth noting are the significantly increased rates of lung cancer associated with divorce or separation and with unemployment. Divorced or separated men and women had higher rates of lung cancer than their married counterparts (rate ratios = 1.34 and 1.83, respectively); as did unemployed men and women compared to their employed counterparts (rate ratios = 1.83 and 2.09, respectively). Relative to married women, women who were divorced/separated, or never married had higher risks of cervical cancer (rate ratios = 1.74 and 1.80, respectively). Incidence rates did not vary significantly by rural–urban residence for any of the cancers examined.

#### Differentials in late-stage cancer diagnosis

Table 6 shows demographic and socioeconomic effects on the likelihood of late-stage cancer diagnoses. The *P*-values are from testing for the overall effect of each demographic and SES characteristic by using the Wald test statistic. The overall test (with more than one degree of freedom) was not a trend test (with one degree of freedom), because we did not assume that the effect of an SES characteristic is linear. Lower income was statistically significantly associated with an increased likelihood of being diagnosed with a late-stage prostate (*P* = 0.002) or breast cancer (*P* = 0.02). For example, men with family incomes less than $12,500 and between $12,500 and $24,999 had elevated odds of late-stage disease compared to men with family incomes ≥$50,000. The odds for late-stage breast cancer for the two lowest income categories are 2.3 and 1.8 times higher than those of the highest income group, respectively. In terms of racial/ethnic differences, the odds of being diagnosed with late-stage prostate cancer for non-Hispanic black males was 2.6 times higher and the odds of being diagnosed with late-stage breast cancer for non-Hispanic black females was 2.2 times higher than their non-Hispanic white counterparts, respectively. The likelihood of a diagnosis of late-stage colorectal cancer did not vary significantly for any of the SES characteristics examined.

## Discussion

Reducing disparities in overall health and in cancer outcomes is a major priority of the U.S. Department of Health and Human Services and of the National Cancer Institute [6]. Reliable data on cancer-related health disparities among socioeconomic and demographic groups is required to set and track the national goals for reducing such disparities. Using data from the SEER-NLMS record linkage study, we have documented for the first time disparities in cancer incidence and late-stage diagnosis by a variety of self-reported individual-level socioeconomic and demographic characteristics for a major segment of the US population. The findings reported here should serve as important baseline statistics for the United States and aid in making future domestic and international comparisons of cancer rates based on individual-level social inequalities in cancer incidence and stage at diagnosis.

The magnitude of individual-level SES disparities in cancer incidence and patient survival shown here may differ from those based on area-level SES data. In the absence of individual socioeconomic information, researchers have often used area-based socioeconomic characteristics of places of residence (e.g., county, zip code, census tract, or block group) appended to cancer and other disease/health records to analyze socioeconomic disparities [23-28]. However, area-based socioeconomic measures are qualitatively and conceptually different from individual-level SES variables [29]. They should not be viewed as proxies for the individual information when the latter is not available. Rather, they should be viewed as community, neighborhood, or social structural influences, which may contribute to individual cancer risks, independently from individual socioeconomic characteristics [29, 30]. We plan in our future studies to employ a multilevel framework to examine both area- and individual-level socioeconomic inequalities in cancer incidence, stage, and patient survival utilizing the SEER-NLMS linked data.

The major findings of this study are generally consistent with the patterns identified in the literature [31-41]. The racial/ethnic patterns in cancer incidence based on this linkage study are generally consistent with those obtained from the cross-sectional SEER data in California for the period 1979–1998 [42]. Significant ethnic and SES disparities in overall cancer incidence were found in the California study, with Asian/Pacific Islanders, Mexicans, and other Hispanics experiencing lower incidence rates and non-Hispanic blacks and those in lower education and income strata experiencing higher rates. However, the magnitude and the direction of the relationship between SES and cancer incidence varied by cancer site and gender. In a study of cancer patients in the San Francisco Bay area SEER registry, the inverse socioeconomic gradients in lung and cervical cancer incidence were particularly pronounced, whereas breast and prostate cancer and melanoma incidence increased substantially with increasing SES [43]. Others have reported socioeconomic patterns in cancer stage that were generally consistent with our study results across the cancers examined; e.g., late-stage diagnosis associated with lower SES [36, 44-46].

Social disparities in cancer incidence may be related to socioeconomic and demographic differences in cancer-related risk factors and behaviors, such as cigarette smoking, poor diet, physical inactivity, obesity, reproductive factors, human papillomavirus (HPV) infection, and sun exposure [31, 47, 48]. Disparities in health care access and use [49], particularly in preventive health services, such as cancer screening [8, 50-52], may contribute to differentials in cancer stage distributions, especially in the late stage diagnosis. Individuals at lower levels of SES, particularly with low educational attainment, are more likely than those with higher education or higher SES levels to be current smokers, to be physically inactive, and to be obese [47]. Marked marital status differentials in cancer incidence may partly reflect differences in SES, behavioral factors [49], social networks, and social support characteristics. More research is needed to determine the causal factors underlying socioeconomic risk gradients, in order to develop innovative and targeted health promotion strategies. For example, Harris [31] noted that smoking behavior was sensitive to price: a tax reform policy may then reduce smoking in low socioeconomic populations, who are most at risk of lung cancer.

Our study is limited by small numbers of cancers diagnosed in some groups. In addition, cancer incidence rates shown in this paper may be underestimated if CPS respondents moved to a non-SEER area and were subsequently diagnosed with cancer. Other limitations of the study include the exclusion of the institutionalized population in the CPS and the time-fixed nature of the covariates over the relatively long cancer incidence follow-up. It is important to point out that socioeconomic characteristics measured closer to the time of cancer diagnosis may be a poor indicator of the effects of socioeconomic position accumulated over the life course [53]. Some characteristics, such as educational attainment is nearly stable or fixed after 25 years of age; while others, such as income [15], marital status, and employment status are more likely to change over time. However, because we used broad family income and occupation categories, the relative impact of any expected changes in social mobility or time-varying covariates should be somewhat minimized. It is also possible that cases matched to the NLMS cohorts are a biased subset of cancer cases identified by SEER Program registries. While analyses of the representativeness of cases included in this study show statistically significant differences, this is not surprising given the large number of cases involved. The magnitude of the differences is small, however, decreasing their epidemiologic importance.

The analytic potential of this linked longitudinal database is not limited to the types of analyses shown here. The database can be used to analyze individual-level variations in site-specific cancer incidence, patient survival, mortality, stage at diagnosis, extent of disease, and treatment by a variety of self-reported characteristics. In addition to the variables we included in our analyses, there are data available from the survey on detailed race/ethnicity, ethnic origin, household size and composition, housing type and tenure, residential mobility, internal migration, veteran status, metropolitan/suburban/non-metropolitan residence, industry, earnings, welfare assistance, labor supply (annual number of hours worked), unemployment duration, availability and type of health insurance coverage, cigarette smoking, and self-assessed health status. In this study we focused on the individual effects of the various socioeconomic factors on cancer rates controlling for age and period of diagnosis, SEER registry area, and sex when relevant. In our future analyses, we will simultaneously examine effects of these factors on cancer outcomes because they may confound with each other.

The SEER-NLMS record linkage study has enabled an evaluation of the quality of demographic data (e.g., race/ethnicity and place of birth) available from medical records and reported by SEER registries as compared with the self-reported data and its impact on health disparity studies [16]. It will also allow multilevel modeling of the effects of area deprivation, environmental factors, health services, and individual socioeconomic status on various cancer outcomes; and assess changing socioeconomic and geographic patterns in cancer incidence, mortality, stage of disease, and survival over time. Moreover, since the SEER-NLMS is being expanded to include additional CPS cohorts and additional cancer patients both from more recent years of diagnoses and from the participation of all SEER registries, the expansion will add greatly to the analytic capability of the linked SEER-NLMS data, which is currently partly limited by its small numbers in certain sociodemographic subgroups. The addition of Medicare enrollment and claims data (from 1990 onward) increases even further the research potential of the linked SEER-NLMS data.

## Figures and Tables

**Table 1:** Comparison of SEER cancer patient demographic characteristics, year of cancer diagnosis, and cancer site between matched cancer patients (used in incidence analyses) and original SEER case file

		SEER cases submitted % (*N*) total cases submitted	Matched cases % (*N*) of matched cases
Total population		100.0 (3,071,661)	100.0 (11,464)
Age group			
	25–34	5.0 (154,918)	0.8 (96)
	35–44	7.5 (229,915)	4.9 (557)
	45–54	11.7 (359,009)	11.1 (1,269)
	55–64	19.9 (611,362)	21.1 (2,418)
	65–74	29.0 (890,605)	32.9 (3,777)
	75–84	20.4 (627,770)	22.8 (2,617)
	85+	6.4 (198,082)	6.4 (730)
Sex			
	Male	48.7 (1,496,772)	52.5 (6,019)
	Female	51.3 (1,574,889)	47.5 (5,445)
Race			
	White	85.6 (2,630,827)	85.7 (9,819)
	Black	8.0 (246,387)	7.2 (824)
	API	4.7 (143,387)	6.3 (718)
	AI/AN	0.2 (4,612)	0.2 (20)
	Other	0.2 (4,795)	0.1 (14)
	Unknown	1.4 (41,653)	0.6 (69)
Ethnicity			
	Non-Hispanic white	79.8 (2,452,160)	79.7 (9,138)
	Non-Hispanic black	7.9 (243,357)	7.1 (814)
	Hispanic	5.4 (165,478)	5.4 (623)
	Others	5.5 (169,867)	7.2 (823)
	Unknown	1.3 (40,799)	0.6 (66)
Registry			
	San Francisco/Oakland	10.1 (310,933)	5.9 (682)
	Connecticut	9.7 (297,011)	8.4 (959)
	Detroit	11.2 (344,754)	11.3 (1,293)
	Hawaii	2.4 (72,967)	6.6 (752)
	Iowa	8.2 (252,294)	11.1 (1,278)
	Seattle	8.8 (271,556)	7.6 (869)
	Utah	2.9 (88,594)	7.9 (906)
	San José/Monterey	2.9 (89,336)	2.2 (253)
	Los Angeles	11.9 (364,961)	11.3 (1,299)
	Greater California	23.1 (709,437)	17.2 (1,968)
	Louisiana	6.3 (192,375)	6.9 (789)
	Kentucky	2.5 (77,443)	3.6 (416)
Year of diagnosis			
	1979–1983	11.0 (339,057)	5.6 (645)
	1984–1988	16.5 (506,413)	15.2 (1,743)
	1989–1993	34.9 (1,071,441)	35.2 (4,037)
	1994–1998	37.6 (1,154,750)	44.0 (5,039)
Cancer site			
	Breast	15.6 (477,812)	14.8 (1,697)
	Prostate	13.4 (411,486)	16.4 (1,881)
	Colorectal	11.6 (357,788)	12.0 (1,375)
	Lung/Bronchus	13.6 (416,522)	14.9 (1,713)
	Cervix	4.8 (147,140)	1.0 (116)
	Melanoma of skin	3.8 (116,850)	2.6 (302)
	Other	37.2 (1,144,063)	38.2 (4,380)

*Source*: SEER_NLMS Record Linkage Study. Based on the 1979 through 1998 follow-up of residents of 11 SEER registries (Iowa, Hawaii, Seattle, Connecticut, Detroit, Utah, Los Angeles, San Francisco/Oakland/San Jose/Monterey, Greater California, Louisiana, and Kentucky) who were 25 years of age or older on their CPS survey date

**Table 2:** Age-adjusted incidence rates^a^, standard errors (SE), covariate-adjusted rate ratios (RR)^b^, and 95% confidence intervais (CI) by selected socioeconomic and demographie characteristics: all cancers combined

Characteristic		All cancers and both sexes combined (*N* = 203,908)						All cancers, male (*N* = 95,964)						All cancers, female (*N* = 107,944)					
		No.	Rate	SE	RR	95% CI		No.	Rate	SE	RR	95% CI		No.	Rate	SE	RR	95% CI	

Total population		11,464	550.21	4.89	–	–	–	6,018	671.00	8.07	–	–	–	5,445	470.89	6.18	–	–	–
Race/ethnicity																			
	Non-Hispanic white	9,068	567.24	5.70	1.00	Reference		4,716	683.06	9.25	1.00	Reference		4,352	494.46	7.35	1.00	Reference	
	Non-Hispanic black	834	671.68	22.28	1.23	1.15	1.32	471	980.95	43.31	1.49	1.35	1.64	363	482.86	24.32	0.98	0.88	1.09
	American Indian/Alaska Native	39	526.99	83.81	0.94	0.69	1.29	15	503.57	130.80	0.76	0.46	1.27	24	523.84	101.04	1.09	0.73	1.63
	Asian/Pacific Islander	661	417.12	15.42	0.74	0.68	0.80	351	480.48	24.04	0.71	0.64	0.79	310	361.91	19.71	0.73	0.66	0.83
	Hispanic	638	416.59	16.47	–	–	–	343	546.77	29.95	–	–	–	295	327.97	18.74	–	–	–
	Mexican	447	428.92	20.57	0.73	0.67	0.81	245	570.27	38.01	0.79	0.69	0.90	202	333.53	23.14	0.67	0.58	0.78
	Other Hispanic	191	396.08	27.95	0.72	0.62	0.83	98	509.60	49.96	0.75	0.61	0.92	93	318.80	32.18	0.67	0.55	0.83
	Other or unknown race	224	587.90	36.84	0.96	0.84	1.09	122	712.60	60.51	0.97	0.81	1.16	102	510.46	47.76	0.98	0.80	1.19
Educational attainment (years of education)																			
	Less than high school graduates (≤11)	3,676	583.64	10.08	1.17	1.10	1.24	2,034	730.30	16.21	1.22	1.13	1.31	1,642	478.52	12.77	1.08	0.98	1.18
	High school graduates (12)	4,084	549.45	8.18	1.14	1.07	1.20	1,906	694.73	14.87	1.17	1.82	1.25	2178	475.34	9.81	1.07	0.98	1.17
	Some post high school education (13–15)	1,847	547.08	12.07	1.11	1.04	1.19	930	657.95	20.36	1.10	1.01	1.20	927	481.38	15.11	1.09	0.99	1.21
	College education or beyond (16+)	1,837	525.47	11.96	1.00	Reference		1,141	602.27	17.22	1.00	Reference		696	443.33	16.53	1.00	Reference	
	Unknown	10	276.03	92.32	0.49	0.26	0.90	7	333.51	151.52	0.58	0.28	1.23	3	191.49	108.34	0.46	0.15	1.43
Family income (1990 dollars)																			
	<$12,500	2,007	568.05	13.43	1.13	1.06	1.20	813	729.50	25.17	1.15	1.06	1.26	1,194	499.84	15.95	1.16	1.06	1.26
	$12,500–$24,999	2,637	568.82	10.86	1.11	1.05	1.17	1373	712.77	18.23	1.14	1.06	1.23	1,264	475.26	13.41	1.08	1.00	1.17
	$25,000–$34,999	1,632	567.27	13.43	1.08	1.02	1.15	906	711.00	21.98	1.13	1.04	1.22	726	461.44	16.75	1.03	0.94	1.13
	$35,000–$49,999	1,773	553.55	12.85	1.03	0.97	1.09	960	634.72	19.73	0.99	0.91	1.07	813	485.35	16.79	1.08	0.99	1.18
	$50,000+	3,067	540.04	10.27	1.00	Reference		1780	637.18	15.67	1.00	Reference		1287	448.57	13.26	1.00	Reference	
	Unknown	348	456.42	24.37	0.88	0.79	0.99	186	554.05	40.36	0.91	0.78	1.06	162	385.65	30.63	0.90	0.76	1.06
Poverty status (ratio of family income to poverty threshold)																			
	At or below 100%	1,158	560.33	16.01	1.12	1.04	1.21	473	723.88	31.20	1.16	1.04	1.30	685	492.52	18.73	1.12	1.05	1.24
	100–200%	2,100	531.20	11.39	1.03	0.97	1.10	1,032	677.48	19.89	1.07	0.98	1.17	1,068	449.80	13.90	1.02	0.93	1.12
	200–400%	3,959	565.37	8.48	1.06	1.00	1.13	2,113	688.76	13.79	1.07	0.99	1.15	1,846	447.90	10.72	1.06	0.97	1.15
	400–600%	2,379	550.63	10.92	1.03	0.96	1.09	1,293	642.85	17.18	1.00	0.92	1.08	1,086	481.04	14.24	1.06	0.97	1.17
	Above 600%	1,868	547.51	12.78	1.00	Reference		1,107	653.04	19.26	1.00	Reference		761	459.62	17.15	1.00	Reference	
Employment status																			
	Employed	5,395	562.59	8.99	1.00	Reference		3,352	645.12	12.87	1.00	Reference		2,043	447.51	11.76	1.00	Reference	
	Unemployed	290	595.70	46.88	1.09	0.97	1.23	186	920.88	74.81	1.16	1.00	1.35	104	418.66	56.25	1.01	0.83	1.23
	Unable to work	255	599.00	40.25	1.17	1.03	1.33	168	793.97	65.06	1.29	1.11	1.51	87	399.65	47.10	0.98	0.78	1.21
	Others/retired	5,511	565.64	8.83	1.19	1.14	1.25	2,301	765.18	25.53	1.20	1.12	1.29	3,210	485.23	9.36	1.12	1.05	1.19
	Unknown	13	261.48	101.72	0.59	0.27	0.78	11	205.68	79.84	0.56	0.31	1.01	2	223.81	148.75	0.45	0.12	1.81
Marital status																			
	Married	8,247	571.56	5.98	1.00	Reference		5,069	716.45	9.39	1.00	Reference		3,354	463.05	7.73	1.00	Reference	
	Widowed	1,435	561.39	33.54	0.94	0.88	1.00	322	861.01	80.57	1.05	0.93	1.18	1,119	523.31	37.75	1.05	0.97	1.13
	Divorced/separated	1,130	579.15	17.45	1.07	1.01	1.14	500	747.80	34.76	1.04	0.94	1.14	658	519.22	20.25	1.11	1.02	1.21
	Never married	637	528.82	21.35	0.96	0.88	1.04	334	605.57	33.53	0.91	0.81	1.02	310	500.68	29.16	1.09	0.96	1.22
	Unknown	15	439.55	127.53	0.56	0.34	0.94	15	791.12	281.18	0.65	0.35	1.20	5	302.02	128.02	0.59	0.24	1.41
Place of residence																			
	Urban	8,928	545.18	5.50	1.00	Reference		4,630	666.83	9.14	1.00	Reference		4,298	467.84	6.92	1.00	Reference	
	Rural	2,526	570.89	10.76	1.04	0.99	1.08	1,380	688.62	17.30	1.03	0.97	1.10	1,146	483.45	13.69	1.03	0.96	1.10
	Unknown	10	334.92	125.63	0.45	0.24	0.84	8	630.57	283.95	0.59	0.30	1.19	2	114.87	79.39	0.32	0.08	1.28

*Source*: SEER-NLMS Record Linkage Study. Based on the 1979 through 1998 follow-up of residents of 11 SEER Registries (Iowa, Hawaii, Seattle, Connecticut, Detroit, Utah, Los Angeles, San Francisco/Oakland/San Jose/Monterey, Greater California, Louisiana, and Kentucky) who were 25 years of age or older on their CPS survey date

a Rates are per 100,000 population and are age-adjusted to the 2000 US standard population by the direct method

b Rate ratios were estimated from Cox regression models that stratified for age at survey and CPS cohort and controlled for sex when relevant

**Table 3:** Age-adjusted incidence rates^a^, standard errors (SE), covariate-adjusted rate ratios (RR)^b^, and 95% confidence intervals (CI) by selected cioeconomic and demographic characteristics: lung cancer

		Lung cancer, male						Lung cancer, female					
Characteristic		No.	Rate	SE	RR	95% CI		No.	Rate	SE	RR	95% CI	
Total population		1,135	116.20	3.38	–	–	–	701	56.77	2.11	–	–	–
Race/ethnicity													
	Non-Hispanic white	895	118.85	3.88	1.00	Reference		575	60.56	2.51	1.00	Reference	
	Non-Hispanic black	104	190.46	18.56	1.73	1.41	2.12	57	72.44	9.63	1.23	0.93	1.61
	American Indian/Alaska Native	2	48.21	33.98	0.55	0.14	2.22	3	80.21	45.91	1.12	0.36	3.50
	Asian/Pacific Islander	60	74.65	9.46	0.65	0.50	0.85	31	35.96	6.03	0.56	0.39	0.81
	Hispanic	51	77.19	11.50	–	–	–	16	20.79	4.61	–	–	–
	Mexican	31	71.38	13.93	0.55	0.38	0.79	9	18.04	5.42	0.25	0.13	0.48
	Other Hispanic	20	94.42	21.60	0.82	0.53	1.29	7	25.75	8.45	0.39	0.19	0.83
	Other or unknown race	23	122.69	25.99	1.00	0.66	1.52	19	92.18	19.14	1.33	0.84	2.13
Educational attainment (years of education)													
	Less than high school graduates (≤11)	493	166.55	7.65	3.01	2.44	3.70	246	71.63	4.91	2.02	1.49	2.73
	High school graduates (12)	385	123.94	6.38	2.32	1.88	2.86	293	59.08	3.36	1.74	1.30	2.35
	Some post high school education (13–15)	141	93.58	7.60	1.67	1.30	2.13	109	56.38	5.34	1.66	1.19	2.31
	College education or beyond (16+)	115	57.60	5.50	1.00	Reference		52	35.91	4.96	1.00	Reference	
	Unknown	1	107.95	102.12	0.81	0.11	5.82	1	62.15	60.86	2.08	0.29	15.07
Family income (1990 dollars)													
	<$12,500	170	150.92	11.95	1.71	1.40	2.09	183	81.44	6.69	1.77	1.40	2.23
	$12,500–$24,999	186	142.75	8.44	1.61	1.36	1.91	174	62.18	4.88	1.40	1.12	1.77
	$25,000–$34,999	196	143.50	9.84	1.60	1.33	1.93	86	50.99	5.49	1.14	0.87	1.49
	$35,000–$49,999	163	93.47	7.53	1.09	0.90	1.33	100	58.79	5.87	1.25	0.97	1.62
	$50,000+	283	90.99	6.06	1.00	Reference		138	45.87	4.15	1.00	Reference	
	Unknown	37	99.31	16.41	1.16	0.82	1.65	20	45.84	10.35	1.06	0.66	1.72
Poverty status (ratio of family income to poverty threshold)													
	At or below 100%	102	151.43	14.94	1.72	1.38	2.27	94	69.76	7.31	1.52	1.13	2.03
	100–200%	227	144.59	9.43	1.67	1.37	2.05	149	62.89	5.33	1.32	1.01	1.73
	200–400%	401	119.60	5.81	1.38	1.15	1.65	239	57.70	3.67	1.23	0.96	1.57
	400–600%	236	105.65	6.92	1.21	0.99	1.47	129	54.35	4.68	1.11	0.85	1.45
	Above 600%	169	90.31	7.25	1.00	Reference		90	47.66	5.09	1.00	Reference	
Employment status													
	Employed	591	10.71	5.05	1.00	Reference		211	55.36	4.48	1.00	Reference	
	Unemployed	50	151.57	25.00	1.83	1.37	2.44	20	75.91	18.64	2.09	1.32	3.31
	Unable to work	44	161.61	26.19	1.93	1.42	2.63	16	80.11	22.71	1.57	0.94	2.64
	Others/retired	448	143.68	10.21	1.42	1.22	1.67	453	65.01	3.34	1.45	1.21	1.73
	Unknown	2	31.47	24.37	0.64	0.16	2.57	1	75.59	73.68	2.15	0.30	15.34
Marital status													
	Married	927	116.25	3.74	1.00	Reference		387	49.09	2.44	1.00	Reference	
	Widowed	50	149.80	32.89	0.96	0.72	1.29	174	82.05	10.73	1.45	1.19	1.76
	Divorced/separated	112	151.35	15.97	1.34	1.10	1.63	120	92.66	8.74	1.83	1.49	2.25
	Never married	46	81.91	12.30	0.77	0.57	1.04	19	30.91	7.48	0.73	0.46	1.16
	Unknown	0	0.00	0.00	0.00	0.00	0.00	1	42.93	42.31	1.07	0.15	7.67
Place of residence													
	Urban	860	114.31	3.82	1.00	Reference		552	56.37	2.35	1.00	Reference	
	Rural	275	123.92	7.36	1.10	0.96	1.27	148	58.26	4.78	1.04	0.86	1.24
	Unknown	0	0.00	0.00	0.00	0.00	0.00	1	58.93	57.77	1.34	0.19	9.53

*Source*: SEER-NLMS Record Linkage Study. Based on the 1979 through 1998 follow-up of residents of 11 SEER Registries (Iowa, Hawaii, Seattle, Connecticut, Detroit, Utah, Los Angeles, San Francisco/Oakland/San Jose/Monterey, Greater California, Louisiana, and Kentucky) who were 25 yearsof age or older on their CPS survey date

a Rates are per 100,000 population and are age-adjusted to the 2000 US standard population by the direct method

b Rate ratios were estimated from Cox regression models that stratified for age at survey and CPS cohort and controlled for sex when relevant

**Table 4:** Age-adjusted incidence rates^a^, standard errors (SE), covariate-adjusted rate ratios (RR)^b^, and 95% confidence intervals (CI) by selected socioeconomic and demographic characteristics: colorectal cancer, prostate cancer, and female breast cancer

Characteristic		Colorectal cancer (both sexes combined)						Prostate cancer						Female breast cancer					
		No.	Rate	SE	RR	95% CI		No.	Rate	SE	RR	95% CI		No.	Rate	SE	RR	95% CI	

Total population		1,467	68.39	1.69	–	–	–	1,995	218.11	4.64	–	–	–	1,739	149.10	3.54	–	–	–
Race/ethnicity																			
	Non-Hispanic white	1,159	69.43	1.93	1.00	Reference		1,561	218.25	5.23	1.00	Reference		1,364	155.18	4.20	1.00	Reference	
	Non-Hispanic black	113	88.54	8.28	1.44	1.30	1.59	188	403.88	29.21	1.87	1.60	2.17	119	153.92	13.67	1.01	0.84	1.22
	American Indian/Alaska Native	5	62.83	28.41	0.99	0.41	2.39	5	226.63	97.82	0.84	0.35	2.03	6	111.41	45.26	0.81	0.36	1.81
	Asian/Pacific Islander	86	54.13	5.69	0.77	0.61	0.95	96	131.82	12.21	0.59	0.48	0.72	111	123.93	11.81	0.82	0.67	0.99
	Hispanic	77	54.79	5.92	–	–	–	110	186.89	17.83	–	–	–	104	106.02	10.57	–	–	–
	Mexican	56	57.58	7.84	0.81	0.62	1.06	79	186.14	21.53	0.87	0.69	1.10	73	107.88	13.17	0.73	0.57	0.92
	Other Hispanic	21	42.40	9.03	0.66	0.43	1.02	31	179.29	30.66	0.75	0.53	1.07	31	103.19	18.09	0.68	0.48	0.97
	Other or unknown race	27	70.38	12.36	1.00	0.68	1.47	35	217.68	36.91	0.91	0.65	1.27	35	170.48	28.27	1.07	0.76	1.51
Educational attainment (years of education)																			
	Less than high school graduates (≤11)	512	71.94	3.26	1.45	1.31	1.61	622	203.50	7.91	0.79	0.70	0.90	407	124.93	6.87	0.74	0.63	0.86
	High school graduates (12)	527	69.50	2.90	1.22	1.04	1.44	592	211.14	8.43	0.83	0.74	0.94	708	151.23	5.65	0.88	0.77	1.01
	Some post high school education (13–15)	217	64.39	4.14	1.13	0.93	1.36	308	221.75	12.19	0.89	0.77	1.03	333	164.23	8.87	0.96	0.82	1.13
	College education or beyond (16+)	211	66.31	4.07	1.00	Reference		471	253.34	11.64	1.00	Reference		290	167.82	9.95	1.00	Reference	
	Unknown	0	–	–	–	–	–	2	125.07	86.81	0.50	0.13	2.01	1	75.65	72.77	0.39	0.06	2.81
Family income (1990 dollars)																			
	<$12,500	286	69.55	4.33	1.20	1.02	1.43	245	201.15	12.81	0.84	0.72	0.98	304	136.35	8.82	0.90	0.77	1.05
	$12,500–$24,999	353	69.63	3.62	1.20	1.02	1.43	430	207.05	9.40	0.87	0.77	0.99	397	152.73	7.89	0.98	0.85	1.12
	$25,000–$34,999	217	72.85	4.78	1.21	1.02	1.43	268	207.64	12.14	0.86	0.74	0.99	225	139.22	9.29	0.87	0.74	1.02
	$35,000–$49,999	208	66.53	4.39	1.05	0.88	1.25	332	220.30	12.11	0.92	0.81	1.05	268	151.15	9.27	0.94	0.81	1.09
	$50,000+	347	64.09	3.51	1.00	Reference		655	232.47	9.58	1.00	Reference		502	158.60	7.50	1.00	Reference	
	Unknown	56	66.74	8.47	1.15	0.86	1.54	65	183.34	22.50	0.79	0.61	1.02	43	104.23	16.76	0.68	0.49	0.93
Poverty status (ratio of family income to poverty threshold)																			
	At or below 100%	157	69.87	5.47	1.24	1.30	1.60	136	209.09	17.06	0.87	0.72	1.00	185	135.48	10.13	0.89	0.73	1.07
	100–200%	280	64.53	3.79	1.11	0.93	1.34	285	177.27	10.04	0.71	0.61	1.06	314	136.93	7.94	0.90	0.76	1.06
	200–400%	525	73.21	3.02	1.21	1.03	1.42	711	230.86	8.09	0.93	0.82	1.05	586	148.28	6.08	0.94	0.88	1.09
	400–600%	291	69.67	3.91	1.10	0.92	1.31	435	212.20	10.10	0.87	0.76	1.00	381	160.76	8.21	1.03	0.88	1.20
	Above 600%	214	63.29	4.09	1.00	Reference		428	236.44	11.31	1.00	Reference		273	157.61	9.66	1.00	Reference	
Employment status																			
	Employed	628	72.36	3.46	1.00	Reference		1,100	229.12	8.02	1.00	Reference		780	149.64	6.17	1.00	Reference	
	Unemployed	38	56.08	9.67	1.29	0.93	1.79	41	224.43	56.36	0.84	0.61	1.15	31	166.74	45.24	0.76	0.53	1.09
	Unable to work	34	83.94	16.43	1.13	0.79	1.60	36	156.74	27.90	0.75	0.54	1.04	14	76.56	24.83	0.51	0.30	0.87
	Others/retired	767	65.31	2.61	1.09	0.95	1.24	815	214.07	9.94	1.00	0.89	1.13	914	146.58	5.37	1.01	0.91	1.13
	Unknown	0	–	–	–	–	–	3	120.53	75.27	0.67	0.22	2.08	0	–	–	–	–	–
Marital status																			
	Married	1,026	70.62	2.06	1.00	Reference		1,687	224.89	5.20	1.00	Reference		1,142	150.29	4.39	1.00	Reference	
	Widowed	225	75.89	13.04	0.97	1.28	1.59	118	229.29	24.00	1.02	0.84	1.23	289	165.94	24.09	1.01	0.87	1.17
	Divorced/separated	132	73.24	6.61	1.08	0.90	1.29	121	187.53	17.83	0.83	0.69	0.99	207	149.80	10.74	0.98	0.84	1.13
	Never married	84	79.46	8.59	1.12	0.89	1.40	64	153.01	17.11	0.63	0.49	0.80	101	157.05	16.69	1.00	0.81	1.23
	Unknown	0	–	–	–	–	–	5	307.62	146.60	1.17	0.48	2.82	0	–	–	–	–	–
Place of residence																			
	Urban	1,159	68.60	1.91	1.00	Reference		1,529	216.42	5.24	1.00	Reference		1,362	147.10	3.95	1.00	Reference	
	Rural	308	68.24	3.65	0.97	0.85	1.10	463	224.75	10.00	1.05	0.94	1.16	377	157.60	7.99	1.06	0.94	1.19
	Unknown	0	–	–	–	–	–	3	278.49	165.49	0.85	0.27	2.66	0	–	–	–	–	–

*Source*: SEER-NLMS Record Linkage Study. Based on the 1979 through 1998 follow-up of residents of 11 SEER Registries (Iowa, Hawaii, Seattle, Connecticut, Detroit, Utah, Los Angeles, San Francisco/Oakland/San Jose/Monterey, Greater California, Louisiana, and Kentucky) who were 25 years of age or older on their CPS survey date

a Rates are per 100,000 population and are age-adjusted to the 2000 US standard population by the direct method

b Rate ratios were estimated from Cox regression models that stratified for age at survey and CPS cohort and controlled for sex when relevant

**Table 5:** Age-adjusted incidence rates^a^, standard errors (SE), covariate-adjusted rate ratios (RR)^b^, and 95% confidence intervals (CI) by selected socioeconomic and demographic characteristics: melanoma and cervical cancer

Characteristic		Melanoma (non-Hispanic white only, both sexes combined)						Cervical cancer					
		No.	Rate	SE	RR	95% CI		No	Rate	SE	RR	95% CI	
Total population (all races/ethnicities)		311	14.92	0.86				110	10.18	1.01			
Race/ethnicity													
	Non-Hispanic white	296	19.18	1.15	–	–	–	71	9.25	1.16	1.00	Reference	
	Non-Hispanic black	–	–	–	–	–	–	14	17.27	4.64	2.00	1.24	3.55
	American Indian/Alaska Native	–	–	–	–	–	–	1	16.38	16.33	2.28	0.32	16.49
	Asian/Pacific Islander	–	–	–	–	–	–	9	10.17	3.40	1.21	0.60	2.42
	Hispanic	–	–	–	–	–	–	15	14.33	3.93	–	–	–
	Mexican	–	–	–	–	–	–	11	15.69	5.15	1.48	1.78	2.83
	Other Hispanic	–	–	–	–	–	–	4	11.82	5.92	1.44	0.52	3.97
	Other or unknown race	–	–	–	–	–	–	0	–	–	–	–	–
Educational attainment (years of education)													
	Less than high school graduates (0–11)	37	12.76	3.02	0.55	0.37	0.82	41	19.50	3.24	3.24	1.68	6.24
	High school graduates (12)	100	17.56	1.78	0.79	0.59	1.07	37	8.77	1.50	1.45	0.75	2.79
	Some post high school education (13–15)	80	26.02	2.91	1.15	0.84	1.58	20	8.88	2.07	1.45	0.71	2.97
	College education or beyond (16+)	79	20.78	2.37	1.00	Reference		12	6.64	2.08	1.00	Reference	
	Unknown	0	–	–	–	–	–	0	–	–	–	–	–
Family income (1990 dollars)													
	<$12,500	22	9.19	2.36	0.59	0.36	0.95	26	15.53	3.26	2.96	1.61	5.43
	$12,500–$24,999	54	16.89	2.48	0.88	0.62	1.24	29	12.69	2.42	2.29	1.27	4.12
	$25,000–$34,999	40	17.37	2.86	0.86	0.59	1.25	14	8.80	2.47	1.48	0.74	2.98
	$35,000–$49,999	66	23.17	2.94	1.17	0.85	1.60	20	10.35	2.49	1.81	0.96	3.39
	$50,000+	102	20.54	2.38	1.00	Reference		19	6.32	1.75	1.00	Reference	
	Unknown	12	26.16	8.18	1.21	0.65	2.24	2	5.60	4.21	1.62	0.27	5.10
Poverty status (ratio of family income to poverty threshold)													
	At or below 100%	12	10.10	3.04	0.54	0.29	1.01	24	17.68	3.66	4.30	1.84	10.06
	100–200%	40	16.18	2.81	0.78	0.52	1.17	29	14.15	2.69	3.35	1.46	7.72
	200–100%	110	20.18	1.95	0.94	0.69	1.29	34	8.99	1.65	1.94	0.86	4.40
	400–600%	71	19.19	2.28	0.92	0.66	1.30	16	7.71	2.18	1.62	0.67	3.95
	Above 600%	63	22.21	3.18	1.00	Reference		7	4.40	1.74	1.00	Reference	
	Employment status												
	Employed	179	20.24	1.66	1.00	Reference		57	9.18	1.28	1.00	Reference	
	Unemployed	5	10.07	4.51	0.60	0.25	1.46	4	10.21	5.30	1.07	0.39	2.95
	Unable to work	3	10.33	6.06	0.75	0.24	2.36	0	–	–	–	–	–
	Others/retired	109	19.09	2.43	1.24	0.91	1.68	49	11.85	1.81	1.24	0.82	1.85
	Unknown	0	–	–	–	–	–	0	–	–	–	–	–
Marital status													
	Married	242	21.80	1.47	1.00	Reference		61	8.54	1.19	1.00	Reference	
	Widowed	15	3.97	1.09	0.55	0.31	0.97	11	17.28	11.34	1.77	0.85	3.69
	Divorced/separated	25	13.83	2.80	0.76	0.50	1.16	22	15.41	3.47	1.74	1.07	2.84
	Never married	14	11.07	3.29	0.50	0.29	0.88	15	14.89	4.58	1.80	1.00	3.22
	Unknown	0	–	–	–	–	–	1	88.14	81.26	6.68	0.91	49.10
Place of residence													
	Urban	230	19.69	1.34	1.00	Reference		86	10.03	1.13	1.00	Reference	
	Rural	66	17.49	2.23	0.90	0.68	1.18	24	10.57	2.17	1.07	0.68	1.69
	Unknown	0	–	–	–	–	–	0	–	–	–	–	–

*Source*: SEER-NLMS Record Linkage Study. Based on the 1979 through 1998 follow-up of residents of 11 SEER Registries (Iowa, Hawaii, Seattle, Connecticut, Detroit, Utah, Los Angeles, San Francisco/Oakland/San Jose/Monterey, Greater California, Louisiana, and Kentucky) who were 25 years of age or older on their CPS survey date

a Rates are per 100,000 population and are age-adjusted to the 2000 US standard population by the direct method

b Rate ratios were estimated from Cox regression models that stratified for age at survey and CPS cohort and controlled for sex when relevant

–, Statistic could not be calculated due to excluded race/ethnic group or zero observations

**Table 6:** Differentials in distant-stage cancer diagnoses among those aged 25+ years at cancer diagnosis by selected baseline socioeconomic and demographie characteristics

		Colorectal cancer (*N* = 1,889)						Prostate cancer (*N* = 2,457)						Female breast cancer (*N* = 2,565)					
Characteristic		No. of distant- stage	Percent	Odds ratio^a^	95% CI		*P*- value	No. of distant- stage	Percent	Odds ratio^a^	95% CI		*P*- value	No. of distant- stage	Percent	Odds ratio^a^	95% CI		*P*- value
					Lower	Upper					Lower	Upper					Lower	Upper	

Total population		388	20.5					227	9.2					142	5.5				
Sex^b^																			
	Male	196	20.1	0.93	0.74	1.17		0.55											
	Female	192	21.1	1.00	Reference														
Race/ethnicity																			
	Non-Hispanic white	298	20.0	1.00	Reference		0.84	153	8.0	1.00	Reference		**<0.001**	104	5.2	1.00	Reference		0.08
	Non-Hispanic black	32	25.0	1.14	0.73	1.77		38	16.2	2.65	1.70	4.13		18	10.3	2.16	1.22	3.80	
	Asian/Pacific Mander	25	20.0	1.32	0.73	2.42		18	15.2	2.14	0.94	4.90		6	3.6	0.87	0.31	2.48	
	Hispanic	21	21.9	1.11	0.65	1.88		9	7.0	1.22	0.58	2.56		8	5.1	1.15	0.52	2.53	
	Other or unknown race^c^	12	22.6	1.27	0.65	2.50		9	12.3	1.83	0.84	3.98		6	7.7	1.65	0.69	3.94	
Educational attainment (years of education)																			
	Less than high school graduates (≤11)	136	22.7	1.48	1.02	2.14	0.23	94	13.7	1.59	1.04	2.42	0.10	44	8.0	1.77	1.01	3.12	0.27
	High school graduates (12)	148	21.0	1.31	0.92	1.87		74	9.6	1.43	0.94	2.19		53	5.2	1.20	0.70	2.05	
	Some post high school education (13–15)	50	18.4	1.10	0.71	1.69		20	5.1	0.88	0.50	1.57		25	5.0	1.21	0.66	2.22	
	College education or beyond (16+)	53	17.0	1.00	Reference			39	6.5	1.00	Reference			20	4.0	1.00	Reference		
	Unknown	1	33.3	2.35	0.21	26.85		0						0					
Family income (1990 dollars)																			
	<$12,500	67	21.0	1.38	0.94	2.01	0.13	40	16.3	2.32	1.40	3.82	**0.002**	30	7.9	2.30	1.31	4.05	**0.02**
	$12,500–$24,999	96	22.9	1.55	1.10	2.18		60	14.0	2.38	1.52	3.71		34	6.1	1.82	1.07	3.10	
	$25,000–$34,999	63	23.1	1.54	1.06	2.24		35	10.7	2.21	1.36	3.60		16	4.6	1.39	0.73	2.63	
	$35,000–$49,999	65	21.4	1.37	0.95	1.98		40	8.7	2.00	1.26	3.18		28	6.4	1.97	1.14	3.41	
	$50,000+	84	17.0	1.00	Reference			42	4.6	1.00	Reference			26	3.4	1.00	Reference		
	Unknown	13	16.5	1.09	0.57	2.10		10	12.8	2.28	1.05	4.95		8	10.8	3.45	1.48	8.03	
Poverty status (ratio of family income to poverty threshold)																			
	At or below 100%	38	21.5	1.33	0.82	2.16	0.60	21	15.4	2.79	1.48	5.27	**0.010**	19	8.4	3.12	1.44	6.76	**0.02**
	100–200%	64	20.8	1.29	0.84	1.96		36	12.3	1.78	1.03	3.09		27	6.7	2.49	1.21	5.14	
	200–400%	130	21.9	1.36	0.94	1.98		83	10.6	2.11	1.33	3.36		48	5.7	2.12	1.09	4.16	
	400–600%	78	20.8	1.27	0.85	1.89		39	7.3	1.51	0.90	2.54		32	6.3	2.37	1.17	4.78	
	Above 600%	49	17.5	1.00	Reference			28	5.2	1.00	Reference			11	2.8	1.00	Reference		
	Unknown	29	18.5	1.04	0.62	1.74		20	11.9	2.31	1.22	4.37		5	2.7	0.87	0.30	2.56	
Employment status																			
	Employed	188	20.0	1.00	Reference		0.48	99	6.1	1.00	Reference		0.07	61	4.8	1.00	Reference		0.42
	Unemployed	13	30.2	1.73	0.88	3.43		4	7.1	1.16	0.40	3.39		5	11.4	2.65	1.00	7.01	
	Unable to work	6	15.0	0.70	0.28	1.70		8	19.1	2.40	1.01	5.69		0					
	Others/retired	180	20.9	1.08	0.82	1.41		114	15.5	1.33	0.95	1.86		76	6.2	1.12	0.76	1.64	
	Unknown	1	25.0	1.37	0.14	13.48		2	16.7	4.76	0.93	24.32		0					
Marital status																			
	Married	260	19.0	1.00	Reference		0.08	178	8.6	1.00	Reference		0.06	81	4.8	1.00	Reference		0.22
	Widowed	52	23.5	1.31	0.91	1.90		16	16.8	1.19	0.65	2.16		26	9.0	1.62	0.98	2.67	
	Divorced/separated	35	22.3	1.18	0.78	1.77		21	13.2	2.19	1.30	3.68		20	7.2	1.59	0.95	2.65	
	Never married	34	29.3	1.78	1.15	2.74		10	9.2	0.95	0.47	1.92		12	4.7	1.16	0.61	2.20	
	Unknown	7	24.1	1.34	0.56	3.20		2	9.5	1.02	0.22	4.82		3	6.5	1.17	0.35	3.93	
Place of residence																			
	Urban	303	20.4	1.00	Reference		0.67	173	9.3	1.00	Reference		0.91	119	5.8	1.00	Reference		0.08
	Rural	84	20.8	1.11	0.83	1.48		54	9.1	1.08	0.75	1.56		22	4.3	0.74	0.46	1.21	
	Unknown	1	33.3	2.07	0.18	23.44		0						1	50.0	15.41	0.91	261.63	

*Source*: SEER-NLMS Record Linkage Study including cancer patients diagnosed from 1973 through 2001 and residing in one of 11 SEER Registries (Iowa, Hawaii, Seattle, Connecticut, Detroit, Utah, Los Angeles, San Francisco/Oakland/San Jose/Monterey, Greater California, Louisiana, and Kentucky) who were 25 years of age or older on their CPS survey date

Bold *P*-value < 0.05, indicating statistical significance

a Odds ratios were estimated from logistic models that controlled for age and period of diagnosis, SEER registry, and sex when relevant. *CI* confidence interval

b SEER variable

c Includes American Indians and Alaska Natives
